# Effects of Biochar-Derived Sewage Sludge on Heavy Metal Adsorption and Immobilization in Soils

**DOI:** 10.3390/ijerph14070681

**Published:** 2017-06-23

**Authors:** Dan Zhou, Dan Liu, Fengxiang Gao, Mengke Li, Xianping Luo

**Affiliations:** 1 Jiangxi Key Laboratory of Mining & Metallurgy Environmental Pollution Control, Jangxi University of Science and Technology, Ganzhou 341000, China; zhoudan1122@163.com (D.Z.); liudanzlld@163.com (D.L.); 15607078809@163.com (F.G.); Limk123456789@163.com (M.L.); 2 Key Laboratory of Ionic-Type Rare Earth Resources Development and Application, Ministry of Education, Ganzhou 341000, China

**Keywords:** biochar, contamination soils, heavy metal, mobility

## Abstract

The object of this study was to evaluate the effect of sewage sludge biochar on adsorption and mobility of Cr, Mn, Cu, and Zn. Biochar (BC400) was produced via pyrolysis of municipal sewage sludge at 400 °C. Maximum adsorption capacities (*q_m_*) for Zn, Cr, Mn, and Cu were 5.905, 5.724, 5.681, and 5.342 mg·g^−1^, respectively, in the mono-metal solution and 2.475, 8.204, 1.01, and 5.415 mg·g^−1^, respectively, in the multi-metal solution. The adsorption capacities for Mn, Cu, and Zn decreased in the multi-metal solution due to competitive adsorption, whereas the capacity for Cr increased. Surface precipitation is an important mechanism in the sorption of these metals on BC400. The 360-day incubation experiment showed that BC400 application reduced metal mobility in contaminated soils, which was attributed to the substantial decreases in the acid-soluble fractions of Cr, Mn, Cu, and Zn (72.20%, 70.38%, 50.43%, and 29.78%, respectively). Furthermore, the leaching experiment using simulated acid rain indicated that the addition of BC400 enhanced the acid buffer capacity of contaminated soil, and the concentration of Cr, Mn, Cu, and Zn in the leachate was lower than in untreated soil. Overall, this study indicates that sewage sludge biochar application reduces the mobility of heavy metal in co-contaminated soil, and this adsorption experiment is suitable for the evaluation of biochar properties for remediation.

## 1. Introduction

Heavy metal contamination of soils represents a serious environmental issue. In China, heavy metal pollution of soils is dramatically increasing under the influence of rapid developments in industry and agriculture [1]. According to the report in the Soil Pollution Condition Investigation Gazette in 2014 [2], agricultural soils in China are mainly polluted with Pb, Cd, Cu, Zn, As, Cr, Hg, and Ni, representing significant risks for agricultural product safety and human health [3,4]. In addition, acid rain and soil acidification, which are long-term environmental problems in southern China, can aggravate the transportation and bioavailability of heavy metals in the soil [5]. Therefore, a number of studies have focused on heavy metal control and remedy in soils, either under laboratory conditions or in situ, and indicated a good effect on different types of contaminated soil restoration when appropriate measures are taken [6,7]. However, as control areas in farmland are generally larger than the contaminated sites, the selection of appropriate techniques is difficult and restricted, because the usage value of land resources needs to be protected and the remediation cost needs to be controlled. Biochar, as a low-cost and environmentally-friendly material, has been used for treating heavy metal pollution in soil [8,9,10].

The addition of biochar to contaminated soils is an effective method to immobilize heavy metals and reduce bioavailability [11,12]. The capacity of biochar to adsorb heavy metals mainly depends on feedstock type and pyrolysis temperature [13,14,15]. A number of studies have described the effect of biomass type (such as agricultural waste, forestry waste, livestock and poultry waste, and municipal sludge) on the adsorption capacities of biochars, especially in terms of heavy metal adsorption; the results showed good application prospects with a significant difference in adsorption capacity of Pb, Cd, Cu, and Zn between different biochars [16,17,18]. Pyrolysis temperature significantly influences pore size distribution, functional groups, elementary composition, and the pH value of biochar [19,20,21], thereby determining adsorption capacity of heavy metal from aqueous solutions and metal mobility in soils [22,23,24]. For example, the high Cr sorption (208 mg·kg^−1^) on sludge biochar in solution has been associated with low pyrolysis temperatures due to the high number of functional organic groups [25]. In contrast, only 32.3 mg·kg^−1^ of Cr has been shown to be immobilized in soil [26]. The removal capacity for Cd (II) and Zn (II) by biochar in solution and soils improved with higher pyrolysis temperatures due to O-containing functional groups, but the effects achieved with liming were more pronounced due to precipitation of Cd or Zn [24]. Based on previous research, the heavy metal adsorption capacity of biochar differs between solutions and soil environments. Compared with solutions, soil systems are extremely complex, making it difficult to demonstrate heavy metal immobility attributed to the biochar. 

The disposal of sewage sludge is a particular concern in domestic wastewater treatment and is linked to significant environmental issues. The process of pyrolysis sewage sludge into biochar is therefore a promising alternative disposal method [27]. In a number of studies, sludge biochar proved to be a suitable material to adsorb metal ions, (i.e., Pb(II), Cd(II), Cr(VI), As(III), Cu(II), Zn(II), and Ni(II)) and remedy contaminated soil [28,29,30]. Lu et al. [31] indicated that sludge biochar could reduce the risk of Cd leaching under acid rain conditions, and the effect of biochar on the reduction of Cd availability is over liming. Méndez et al. [32] found that sewage sludge biochar significantly decreased the leaching risk of Cu, Zn, Ni, Cd and plants availability compared with raw sewage sludge. Biochar therefore represents a cost-appropriate and environmental friendly approach to the disposal and reuse of sewage sludge.

Although adsorption experiments are commonly carried out to optimize biochar preparation conditions, it remains unclear whether a selected biochar can effectively be used for soil remediation. Therefore, more investigations are needed to evaluate the relationship between biochar properties and heavy metal immobilization capacity. In this study, sludge biochar produced under optimal conditions was used to evaluate the capacities of adsorbing Cr, Mn, Cu, and Zn in solution. Furthermore, a 360-day incubation experiment and a leaching experiment were carried out to determine the effect of biochar on immobility of Cr, Mn, Cu, and Zn in contaminated soils. These results were assessed to determine the application of sewage sludge biochar as a soil amendment. 

## 2. Materials and Methods

### 2.1. Soil Preparation and Characterization

Samples of unpolluted soil (depth 0–30 cm) were collected from an agricultural area in Ganzhou City, Jiangxi province, China. The soil type was Alumi-Plinthic Acrisols according to the FAO soil classification [33]. The physical and chemical soil properties are shown in Table 1. Solutions of K_2_Cr_2_O_7_, MnSO_4_·H_2_O, CuSO_4_·5H_2_O, and ZnSO_4_ were combined with the soil to obtain Cr^6+^, Mn^2+^, Cu^2+^, and Zn^2+^ concentrations of 100, 100, 50, and 250 mg·kg^−1^, respectively. The synthetic ‘contaminated’ soils were then sieved through a 2-mm mesh, placed in plastic containers, and cultured for one month at room temperature for subsequent experiments. The pH was measured in a soil:deionized water suspension at a ratio of 1:2.5 (*w/v*) using a digital pH meter (PHS-3C, Leici, Shanghai, China). Soil organic matter was measured via potassium dichromate oxidation-ammonium ferrous sulfate titration [34]. Cation exchange capacity was analyzed using the EDTA-acetic acid ammonium salt exchange method [35]. The Cr, Mn, Cu, and Zn contents in soil were analyzed by flame atomic adsorption spectrophotometry (FAAS) (SN-SF8, Puxin, Beijing, China) after mixed-acid digestion (HCl-HNO_3_-HClO_4_-HF) using a graphite furnace digester (JRY-X350-24, Hunan, China) according to the EPA 3052 [36] methods.

### 2.2. Biochar Preparation and Characterization

Sewage sludge (SS) for the production of biochar was obtained from the Shang You Municipal Sewage Treatment Plant in Jiangxi, China. The sludge was air-dried at room temperature, placed in a corundum crucible, and pyrolyzed in a muffle furnace (FO810C, Yamato Scientific, Chongqin, China) under nitrogen atmosphere. Pyrolysis temperature was 400 °C for 2 h, at a heating rate of 10 °C·min^−1^. Subsequently, the pyrolyzed biochar (BC400) was cooled down to room temperature, passed through a 0.2-mm mesh sieve, and stored prior to experiments. The characteristics of SS and BC400 are shown in Table 1. Biochar pH was determined in a soil:deionized water suspension at a ratio of 1:10 (*w/v*) using a digital pH meter (PHS-3C) after shaking for 30 min [37]. Moisture and ash contents were determined according to the procedures ASTM D2867-09 (2014) [38] and ASTM D2866-11 [39], respectively. Biochar contents of C, H, O, and N were determined using an elemental analyzer (MicroCube, Elementar, Germany). Brunauer-Emmett-Teller (BET) surface area was determined with N_2_ (at 77 k) adsorption isotherm, using an ASAP 2460, Micromeritics Instrument Corp., Norcross, GA, USA). Fourier-transformed infrared (FTIR) spectra were measured in a KBr pellet in the 4000–400 cm^−1^ region at 4 cm^−1^ by using a Spectrum GX spectrometer (Nicolet is5, Thermo Nicolet Corporation, Waltham, MA, USA). The crystal structure of biochar were measured witha X-ray diffraction (XRD) (RigakU Miniflex, Tokyo, Japan), and analyzed by MDI Jade 5.0 software (Materials Data Inc., Livermore, CA, USA). Total contents of Cr, Mn, Cu, and Zn in SS and BC400 were also measured using the EPA 3052 methods [36].

### 2.3. Sorption Experiments

We used solutions with Cr^6+^, Mn^2+^, Cu^2+^, and Zn^2+^ concentrations from 5 to 100 mg·L^−1^ for adsorption isotherm experiments. Briefly, 1 g of BC400 was added to 100 mL of a heavy metal solution in a 250-mL conical flask and shaken for 24 h at 25 ± 1 °C and 200 r·min^−1^ in a thermostatic oscillator. Then the suspensions were removed, centrifuged at 3000 r·min^−1^ for 15 min, and filtered through a 0.45-μm membrane filter. Concentrations of Cr^6+^, Mn^2+^, Cu^2+^, and Zn^2+^ were determined using flame atomic absorption spectrophotometer (FAAS). Adsorption capacity (*q_t_*) and removal rate (*r*) were calculated according to [40]:

(1) $$q_{t}=\left(c_{0}-c_{t}\right)\;\times \;V/m$$

(2) $$r=\frac{\left(c_{e}-c_{0}\right)}{c_{0}}\times 100\%$$

where *q_t_* is the adsorption amount of heavy metals (Cr^6+^, Mn^2+^, Cu^2+^, and Zn^2+^) at time *t* (mg·g^−1^), *m* is the weight of biochar (g), *V* is the volume of the solution (L), *c_0_* is the initial heavy metal concentration, *c_t_* is the heavy metal concentration at time *t* in solution, and *r* is the adsorption efficiency of heavy metals on BC400.

The mathematical equations of adsorption models with the Langmuir and Freundlich (LM and FM) terms were used to analyze the sorption of Cr, Mn, Cu, and Zn onto BC400, according to Equations (3) and (4):

(3) $$q_{e}=\frac{bq_{m}c_{e}}{1+bc_{e}}$$

(4) $$q_{e}=kc_{e}^{\left(1/n\right)}$$

where *c_e_* is the equilibrium aqueous concentration of heavy metal (mg·L^−1^) and *q_e_* is the equilibrium adsorbed concentration of heavy metal (mg·g^−1^). The parameters *b* and *k* are the adsorption coefficients of LM and FM (L·mg^−1^) and ((mg·g^−1^)(mg·L^−1^)^−n^), respectively. The factor *q_m_* is the maximum adsorption capacity of the solute (mg·g^−1^) and *n* is the Freundlich constant related to the surface site heterogeneity.

### 2.4. Leaching Experiment and Speciation of Heavy Metals

We added BC400 with mass ratios 5% (*w/w*) to the synthetic contaminated soils and incubated the mixtures in pots at room temperature under natural ventilation. Moisture was kept at 60% of maximum field water holding capacity. Soil samples were collected every 3 months, air-dried, and grinded to pass through a 0.2-mm mesh sieve for speciation analysis of heavy metal, following the procedure of the Community Bureau of Reference (BCR) [41,42]. The extracted solutions were filtered through a 0.45-μm filter and heavy metals were determined via FAAS.

**Figure 1:**
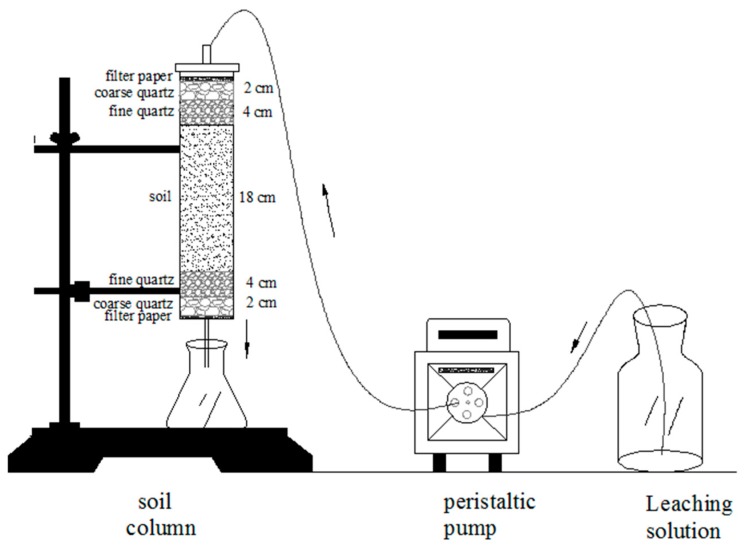
Equipment for the leaching experiment.

A column leaching experiment was performed with plexiglass columns (30 cm height, 5 cm inner diameter) to assess the effect of biochar on mobility of metals (Cr, Mn, Cu, and Zn) in the soil. The inner column wall was processed as a rough surface to avoid preferential lateral flow pathways; during filling, the suspension was stirred several times to prevent the formation of a soil layer. The columns were packed with 100 g of BC400 remediated soil (incubated for 360 days), as control experiment, untreated-contaminated soilwere packed in the another column. The soil layer was 18 cm, with 2 cm layer of quartz sand and 4 cm layer of quartz sand both on the top and bottom of the column (Figure 1). Soil in columns was slowly saturated with deionized water from the bottom of the column before leaching. The solution of stimulated acid rain (SAR, pH 4.5) as a leaching agent was prepared using a mixture of H_2_SO_4_ and HNO_3_ (6:1 mol·mol^−1^) according to the chemical composition of acid rain in Jiangxi Province [43]. Deionized water was used as a negative control. The chemical properties are shown in Table 2 and Table 3. Leaching solutions were added from the top of the column using a peristaltic pump at a flow rate of 23 mL·h^−1^, with an interval of 24 h between each 100 mL. Leachate was collected in conical flasks and the leaching process was terminated after 20 days, when the total leachate volume reached 2000 mL. Leachate pH was determined using a digital pH meter (PHS-3C) and heavy metal contents were measured by FAAS after filtering through a 0.45-μm membrane filter.

### 2.5. Statistical Analysis

All analyses were performed in triplicate, with an experimental error below 5%. Analyses were performed using Origin Pro 8.5 (OriginLab Corporation, Northampton, MA, USA) and the software package SPSS19.0 (IBM SPSS, Armonk, NY, USA).

## 3. Results and Discussion

### 3.1. Adsorption Isotherms

**Figure 2:**
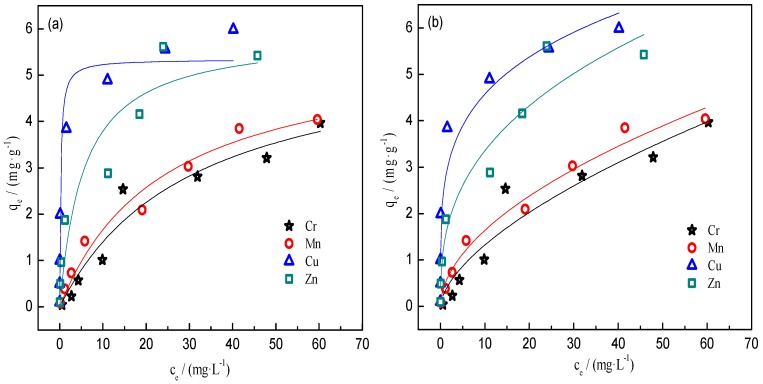
Sorption isotherms of heavy metals onto BC400. ☆ Cr; ○ Mn; △ Cu; □ Zn. (**a**) Langmuir model fitting curve; (**b**) Freundlich model fitting curve.

Adsorption isotherms of Cr, Mn, Cu, and Zn onto BC400, using the Langmuir and Freundlich models, are illustrated in Figure 2, and the fitting parameters of the models are shown in Table 4. For Cr and Cu, adsorption was better fitted to Langmuir equations, indicating mainly monolayer adsorption [44]. In contrast, for Mn and Zn, adsorption was better fitted to Freundlich equations. Though the R^2^ coefficients for Mn according to the Langmuir and Freundlich models were comparable, all nonlinearity values in the Freundlich model were higher than 1.0, indicating that Cr, Mn, Cu, and Zn initially bind onto high energy sites of BC400, followed by lower energy sites [45], and the adsorption of Mn was mainly monolayer adsorption. According to the Langmuir model, the maximum adsorption capacities (*q_m_*) for Zn, Cr, Mn, and Cu were 5.905, 5.724, 5.681, and 5.542 mg·g^−1^, respectively. Ionic radius is one of the main parameters affecting sorption [46]. Our study result disagrees with the findings of Houben et al. [47] and Bogusz et al. [48], who reported greater biochar adsorption capacities with decreasing ionic radius values. In our study, Zn had the largest radius (0.74 Å), combined with the strongest adsorption capacity. In the Langmuir model for Cu, the sorption isotherm was steeper at the beginning (Figure 2) and the *b* values were greater (Table 4). This indicates stronger Cu^2+^ adsorption on BC400, which was considered to qualitatively reflect the affinity between Cu^2+^ and the carboxyl groups of BC400 containing elements (such as S, N, O, and P) with unshared pairs of electrons [44]. The maximum adsorption capacity for Cu on BC400 in this study was higher than that of other biochars (such as softwood-derived biochar [49], beech wood chips, garden green waste residues-derived biochar, and granulated activated carbon [18]), indicating that BC400 is an effective sorbent for the removal of heavy metals from wastewater.

**Figure 3:**
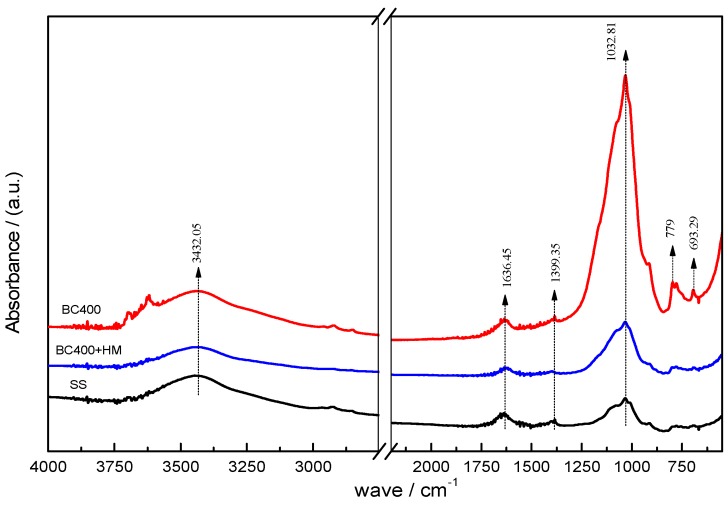
Fourier-transformed infrared spectra (FTIR) of SS and BC400 before and after adsorption.

**Figure 4:**
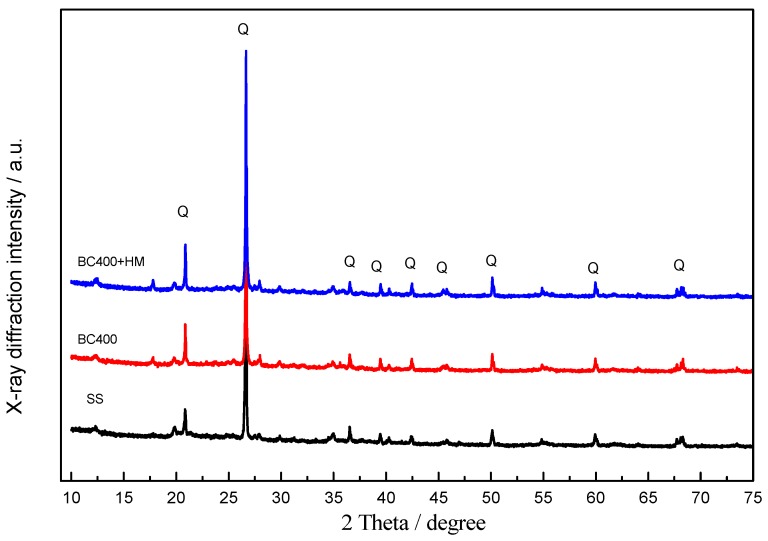
X-ray diffraction (XRD) patterns of SS and BC400 before and after adsorption; Q represents SiO_2_.

The adsorption of metal ions from aqueous solutions is generally governed by the surface chemistry of the sorbent or by precipitation reactions [50,51]. Changes in the functional groups in the sludge and biochar are shown in Figure 3, using FTIR spectra. The peak at 1032 cm^−1^ was assigned to the C–O stretching vibration [52]. Compared with the sewage sludge sample, the peak was stronger in the BC400 sample, mainly due to the loss of alkyl groups during the pyrolysis process. For BC400 in our study, the new peaks after pyrolysis at 779 and 693.29 cm^−1^ can be attributed to out-of-plane bending vibrations of aromatic C–H groups and olefin, respectively [53], indicating that pyrolysis enhanced the degree of aromatization. In contrast, the peak at 3432.05 cm^−1^ was mainly due to the stretching vibration of O-H of alcohols and phenol by intermolecular hydrogen bonds, while the peak at 1636.45 cm^−1^ was associated with the aromatic C=O stretching vibration and the C=C stretching vibration of carboxylic acids esters, ketones, and anhydrides [52]. The peak at 1399.35 cm^−1^ can be assigned to C–H or –CH_3_ bending [54]. These peaks indicate the existence of –CH, –OH, and –COOH on the surface of sewage sludge and pyrolysis at 400 °C only slightly affected these surface functional groups. The FTIR spectrum of BC400 with adsorbed Cr, Mn, Cu, and Zn is presented in Figure 3. It is obvious that the peak at 1032.81 and 693.29 cm^−1^ became weaker, while the split peak at 779 cm^−1^ disappeared. This was likely the result of the coordination between the organic and inorganic functional groups and Cr, Mn, Cu, or Zn; the precipitation process might take place on the surface of BC400. Oxygen-containing functional groups play an important role in the adsorption process [55], which was confirmed in our study via XRD analysis. The XRD patterns of BC400 before and after heavy metal adsorption are presented in Figure 4, showing inorganic minerals, such as SiO_2_ and Al/Si oxides, in the sewage sludge, with the typical 20.92°, 26.64°, 27.92°, 39.45°, 42.4° in the 2θ degree. After reaction with Cr, Mn, Cu, and Zn, these peaks became stronger, implying that precipitates were formed during sorption [56] and suggesting that surface precipitation is an important sorption mechanism for these metals.

### 3.2. Effect of Co-Existence of Cr, Mn, Cu, and Zn on the Adsorption Capacities of BC400

**Figure 5:**
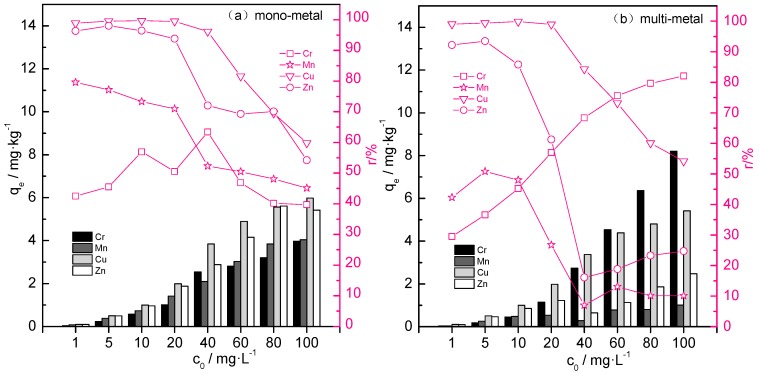
Adsorption efficiency of BC400 for heavy metals in mono-metal and multi-metal solution. □ Cr; ☆ Mn; ▽ Cu; ○ Zn. (**a**) in mono-metal solution; (**b**) in multi-metal solution.

The efficiency of BC400 to remove Cr, Mn, Cu, and Zn in mono- and multi-metal solutions is illustrated in Figure 5. Based on our results, efficiency was higher in the mono-metal compared to the multi-metal solution. The removal efficiency of metals in the mono-metal solution was higher than the multi-metal solution; the maximum removal rate reached 99.63% of Cu, 98.06% of Zn, and 79.6% of Mn in the mono-metal solution, and 98.85% of Cu, 93.5% of Zn, and 50.78% of Mn in the multi-metal solution. Maximum Mn removal decreased in multi-metal solutions relative to mono-metal solutions, while the removal efficiency of Cr generally increased with increasing metal concentrations, reaching 82.04%. This indicates competitive adsorption processes in multi-metal solutions, and the capacities for the four metals Cr, Cu, Zn, and Mn were 8.204, 5.415, 2.475, and 1.01 mg·g^−1^, respectively. Such competitive sorption processes for surface adsorption sites have also been described in previous studies [57,58,59]. The competitive capability strongly depends on the metal contaminant and the biochar type [60].

In our study, the performance of the sludge biochar and its sorption capacity might be the result of various processes on the carbon surface, such as: (I) ionic exchange between metal cations and ionizable protons; (II) precipitation as insoluble matter, such as carbonate and phosphate minerals; and (III) complexation with free carboxyl functional groups, hydroxyl functional groups, and oxygen-containing groups [19,40].

### 3.3. Effects of Biochar on Heavy Metal Mobility in Contaminated Soil

**Figure 6:**
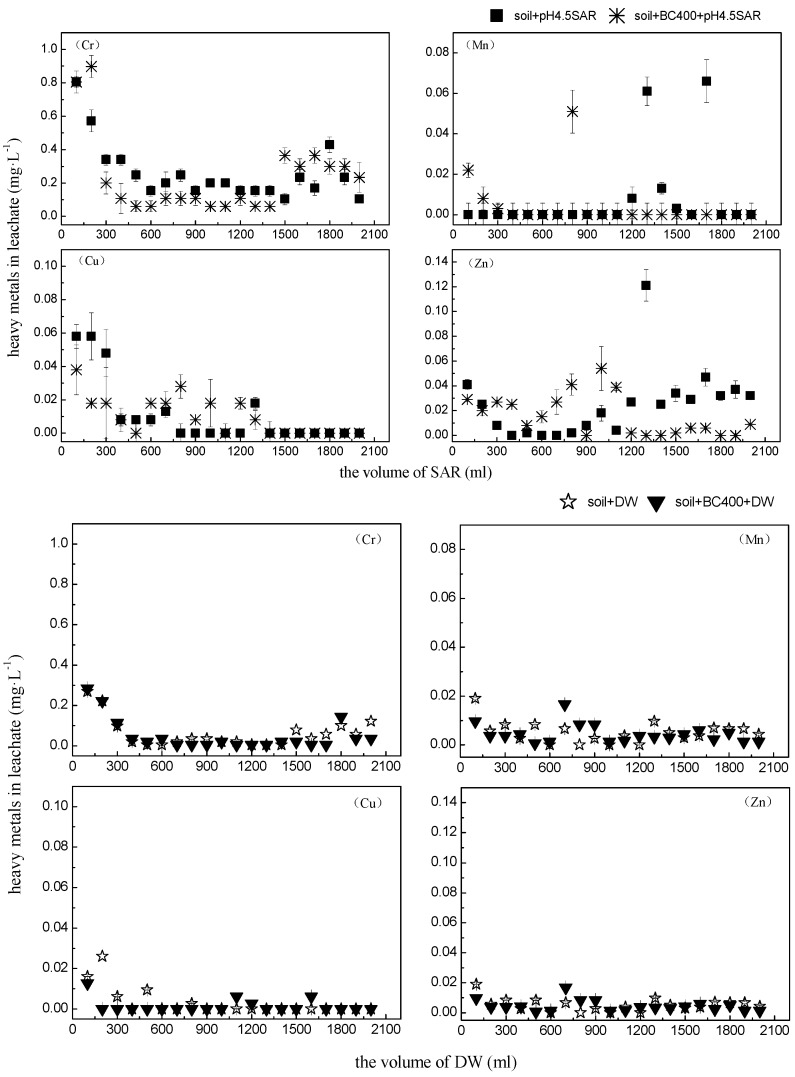
The content of heavy metals in leachate. ■ soil + SAR; ＊ soil + BC400 + SAR; ☆ soil + DW; ▼ soil + BC400 + DW. SAR, simulated acid rain.

**Figure 7:**
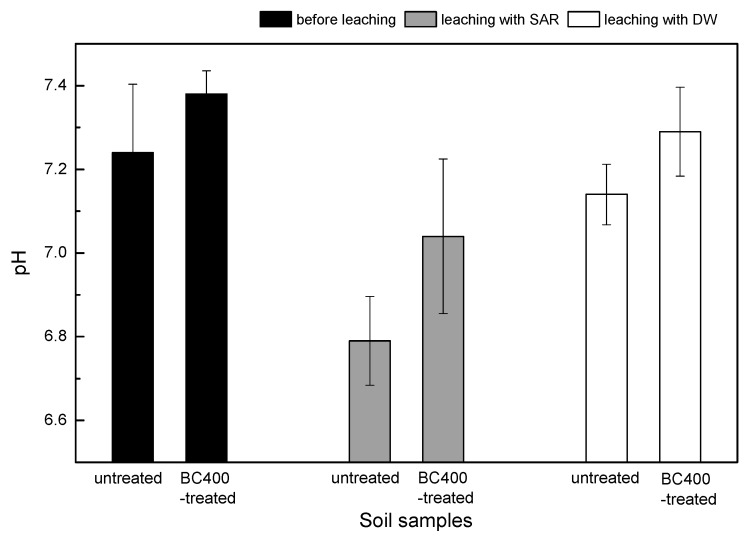
Changes of soil pH before and after leaching.

Several studies have evaluated the effects of biochar on heavy metal mobility. According to the results, biochar can enhance the soil pH and thereby reduces the mobility and availability of Pb, Cd, Cu, and Zn in contaminated soils [11,47]. Soil acidification and acid rain have been suggested to be the most important factors for metal mobilization in contaminated soils, increasing the risk of metals leaching into the ecosystem [5,61]. The authors of previous studies in this field reported a higher pH buffering ability of soils amended with biochar compared with limed soils, as well as decreased Cd mobility and leaching [8,31]. In our study, the accumulated amounts of the four metals in the leachate are shown in Figure 6. We observed lower levels of accumulated metals in the leachate from soil with BC400 compared with untreated soil after SAR solution (pH 4.5) leaching; the average concentrations of Cr, Mn, Cu, and Zn in leachate from untreated soils were 0.2595 mg·L^−1^, 0.0076 mg·L^−1^, 0.011 mg·L^−1^, and 0.0246 mg·L^−1^, respectively, while the average concentration in the leachate from BC400-treated soils were Cr (0.2328 mg·L^−1^), Mn (0.0042 mg·L^−1^), Cu (0.0099 mg·L^−1^), and Zn (0.0155 mg·L^−1^). A low metal concentration in the leachate indicates lower mobility in the soil, confirming the efficiency of BC400, albeit with the effects differing between metals [35]. We observed heavy metal contents increasing in the following order: Mn < Cu < Zn < Cr. This indicates that the effects of BC400 on metal mobility are inconsistent with the adsorption capacities of biochar for Cr, Mn, Cu, and Zn and emphasizes the need for further research. In the controlled experiment, the metal concentrations in the leachate of deionized water (DW) were negligible in both treatments (BC400 and untreated control), indicating that acid rain increases the risk of heavy metal leaching. Two abilities of BC400 impact heavy metal mobility in the soil: Firstly, a large specific surface area, porous structure, and a high number of functional groups on the surface facilitate the formation of insoluble clathrates with heavy metal ions through chelation and complexation processes [62]. Secondly, the higher soil pH and the high buffering capacity increase resistance to acid rain; positive ions of soil heavy metals gradually transform to hydroxyl, increasing the number of soil adsorption sites [63]. A similar study has found that BC400 addition can immobilize heavy metals in the soil due to increasing pH levels, thereby decreasing leaching [31]. The pH change in untreated and BC400-treated soils before and after leaching are presented in Figure 7; the pH in the BC400-treated soil was 0.14 higher than that in the untreated soils with the 360-day incubation before leaching. After leaching with SAR, the pH decreased by 0.45 in untreated soils and by 0.34 in BC400-treated soils, confirming the hypothesis that biochar can enhance the soil pH and the buffering capacity, thereby increasing the resistance to acid rain [64]. These effects are mainly due to the dissolution of alkaline components (such as OH^−^, SiO_3_^2−^, and CO_3_^2−^) in the soil [65,66].

### 3.4. Effects of Biochar on Heavy Metal Speciation in Contaminated Soils

**Figure 8:**
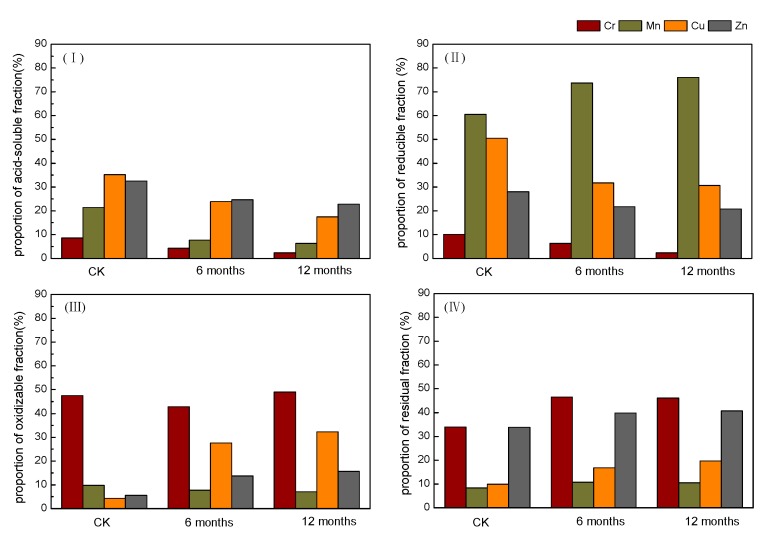
Variation of heavy metal speciation over time with BC400 addition. (**I**) acid-soluble fraction, (**II**) reducible fraction, (**III**) oxidizable fraction, (**IV**) residual fraction). CK, untreated soils.

The speciation of heavy metals in soils is a significant criterion for transfer and bioavailability and was therefore investigated in this study. The BCR sequence includes the acid-soluble, reducible, oxidizable, and residual fractions of the metals, of which the acid-soluble fraction is regarded as the main mobile and bioavailable form [67], representing a greater risk for the surrounding environment. Reducible and oxidizable fractions are considered slow release fractions and can transform to acid-soluble species under acid conditions, whereas the residual fraction is relatively immobile with a low bioavailability; it is fixed in the soil lattice and hardly used by organisms [68]. The results of the fraction analysis are illustrated in Figure 8. After 360 days (12 months) of incubation, compared with the untreated soils (CK), the acid-soluble fractions of Cr, Mn, Cu, and Zn in amended soil significantly decreased by 72.20%, 70.38%, 50.43%, and 29.78%, respectively, but the residual fraction of Cr, Mn, Cu, and Zn increased by 26.47%, 20.94%, 49.63%, and 16.8%, respectively. Meanwhile, the change of reducible and oxidizable fractions showed a different trend. The reducible fractions of Cr, Cu, and Zn in BC400-treated soil decreased 76.39%, 39.41%, and 25.82%, respectively. However, reducible Mn increased by 20.43% by the end of the incubation because the reducible Mn and amorphous Fe commonly exist in a combined form in soil [69]. The oxidizable fractions of Cu and Zn significantly increased by 86.52% and 64.20% in BC400-treated soil, respectively, oxidizable Cr only slightly increased, and oxidizable Mn showed no overall change. After the 360-day incubation, the main fraction of Cr was oxidizable and residual in biochar-treated soil in accordance with CK, and reducible fraction represented the majority of Mn in accordance with CK. However, the main fraction of Cu changed dramatically between BC400-treated soil and CK; acid-soluble and reducible fractions were the main components in CK, but the oxidizable fraction represented a higher proportion in soil containing biochar. The acid-soluble and residual Zn were the main fractions in CK, whereas the residual fraction had the higher proportion due to the decrease of the acid-soluble fraction in soil with biochar. The speciation variations of the four metals, especially the decreases in the acid-soluble fractions, further illustrated that BC400 effectively stabilizes Cr, Mn, Cu, and Zn, which can be attributed to increased soil pH values and adsorption of biochar [70]. 

**Figure 9:**
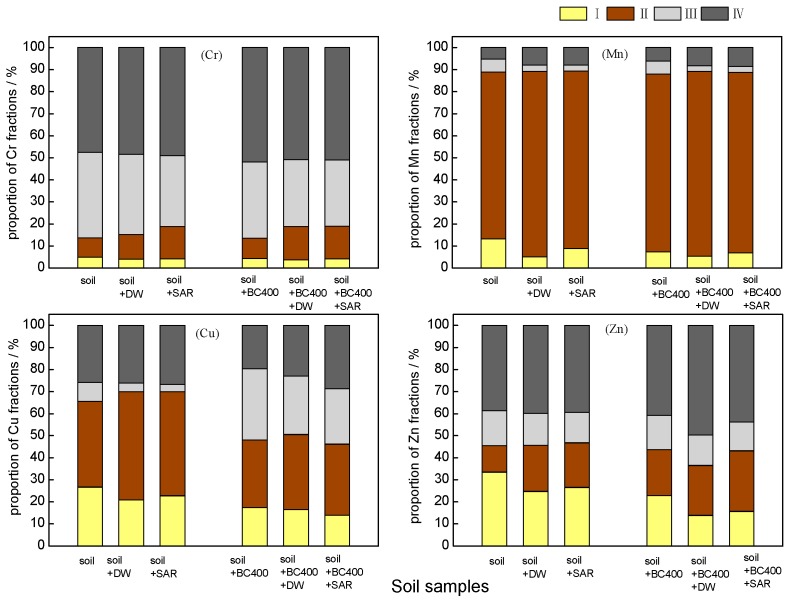
The effect of leaching process on the fractions of heavy metal. (**I**) acid-soluble fraction, (**II**) reducible fraction, (**III**) oxidizable fraction, (**IV**) residual fraction). CK, untreated soils.

After leaching, the proportions of the acid-soluble fractions of the four metals decreased with SAR, both in BC400-treated soils and in untreated soils (Figure 9). In BC400-treated soils, Cr, Mn, Cu, and Zn decreased by 0.28%, 0.59%, 3.39%, and 6.58%, respectively, whereas in untreated soils, the metals decreased by 0.72%, 4.5%, 4.02%, and 6.95%, respectively. The acid-soluble fraction in BC400-treated soils was lower than that in untreated soils due to the acid buffering capacity of biochar. Levels of Cr, Mn, Cu, and Zn associated with the oxidizable fraction significantly decreased by 12.78%, 53.8%, 22.47%, and 17.24%, respectively, with the BC400 treatment. In contrast, the oxidizable fraction-bound Cr, Mn, Cu, and Zn in untreated soils decreased by 17.27%, 54.88%, 60.65%, and 12.1%, respectively, suggesting that SAR leaches the acid-soluble and oxidizable fractions. As a control test, the leaching efficiency of deionized water, for the acid-soluble fractions of Cr, Mn, Cu, and Zn, can be ignored. The results of the leaching experiment show that biochar addition can improve the acid buffering capacity of soil and further reduce the available fractions of heavy metals in soil. In addition, acid rain aggravates the available fractions of heavy metals in soil, and increases the mobility of heavy metals in the long term [71]; the application of biochar slows the effects of acid rain.

## 4. Conclusions

The BC400 biochar can efficiently remove metals from wastewater. Of the studied heavy metals, Cr and Cu fit better with Langmuir model equations, whereas the Freundlich model best described the equilibrium sorption data for Mn and Zn. The maximum adsorption capacities of BC400 for the four metals followed the order Zn > Cr > Mn > Cu in mono-metal solutions and Cr > Zn > Cu > Mn in multi-metal solutions. Overall, the removal efficiency of BC400 was higher in mono-metal solutions than in multi-metal solutions. Surface precipitation is a major mechanism in Cr, Mn, Cu, and Zn sorption on BC400, with competitive sorption between the metals. The incubation experiment confirmed that BC400 application in co-contaminated soils can slightly increase soil pH, thereby changing the speciation of Cr, Mn, Cu, and Zn and increasing metal stability. Under simulated acid rain conditions BC400 had a positive effect on Cr, Mn, Cu, and Zn immobilization, thus reducing the risk of heavy metal losses to the environment. The acid-soluble and oxidizable fractions of four metals were most prone to leaching. The results indicate that it is feasible to amend metal contaminated soils with sewage sludge biochars, which reduce the mobility of metals and leaching risks. Although there are inconsistencies between the adsorption capacities of biochar for Cr, Mn, Cu, and Zn in solution and the stabilization in soil, adsorption experiments represent an effective method to evaluate the suitability of optimized biochar for soil remediation.

## Figures and Tables

**Table 1:** Main characteristics of sewage sludge (SS) and its biochar (BC400).

Property	Soil	SS	BC400
Sand (%)	12	-	-
Silt (%)	56	-	-
Clay (%)	32	-	-
pH (H_2_O)	7.89	6.63	6.42
Total C (wt %)	-	17.67	11.79
Total H (wt %)	-	3.201	0.79
Total O (wt %)	-	21.79	8.04
Total N (wt %)	-	3.17	1.04
H/C	-	2.17	0.8
O/C	-	0.92	0.51
Ash (wt %)	-	56.81	73.39
Yield (%, *w/w*)	-	-	57.63
organic matter (g·kg^−1^)	20.396	50.31	11.32
Moisture content (%)	3.75	23.92	0.14
Cation exchange capacity (cmol·cm^3^·kg^−1^)	2.83	34.9	21.1
BET Surface area (m^2^·g^−1^)	-	1.9687	17.3589
Average pore size (nm)	-	13.39247	8.85609
Pore volume (cm·g^−1^)	-	0.006591	0.038433
Micropore volume (cm^3^·g^−1^)	-	0.000354	0.001471
Total K (mg·kg^−1^)	525.4	67.75	75.12
Total Cr (mg·kg^−1^)	217	36.9	40.25
Total Mn (mg·kg^−1^)	301.4	254.4	264.3
Total Cu (mg·kg^−1^)	54.7	100.1	101.6
Total Zn (mg·kg^−1^)	53.9	12.6	21.35

**Table 2:** Negative and positive ions in precipitation in Jiangxi Province (mmol·L^−1^).

Ions	SO_4_^2−^	NO_3_^−^	F^−^	Cl^−^	NH_4_^+^	Ca^2+^	Mg^2+^	Na^+^	K^+^
Average value of three years	0.206	0.024	0.014	0.025	0.05	0.148	0.019	0.014	0.01

**Table 3:** The chemical composition content in the base of simulated acid rain (mg·L^−1^).

Component	CaSO_4_·2H_2_O	MgSO_4_·7H_2_O	NaF	KCl	(NH_4_)_2_SO_4_	NH_4_NO_3_	HCl
Concentration	25.456	4.674	0.588	0.745	5.07	0.869	0.548

**Table 4:** Parameters of the Langmuir (LM) and Freundlich (FM) isotherm models for heavy metal adsorption onto sludge biochar.

Heavy Metal	LM			FM		
	*b*	*q_m_*	*R^2^*	*k_F_*	*n*	*R^2^*
Cr	0.0324 ± 0.014	5.724 ± 1.141	0.9402	0.321 ± 0.126	1.627 ± 0.290	0.9172
Mn	0.041 ± 0.013	5.681 ± 0.723	0.9727	0.475 ± 0.084	1.861 ± 0.170	0.9797
Cu	4.498 ± 1.599	5.342 ± 0.279	0.9554	2.671 ± 0.294	4.288 ± 0.650	0.9327
Zn	0.179 ± 0.127	5.905 ± 0.981	0.8908	1.441 ± 0.284	2.727 ± 0.456	0.9380
